# SARS-CoV-2 anti-N antibodies among healthcare personnel without previous known COVID-19

**DOI:** 10.1017/ash.2024.389

**Published:** 2024-10-22

**Authors:** Sajal Tiwary, Caroline A. O’Neil, Kate Peacock, Candice Cass, Mostafa Amor, Meghan A. Wallace, David McDonald, Olivia Arter, Kelly Alvarado, Lucy Vogt, Henry Stewart, Daniel Park, Victoria J. Fraser, Carey-Ann D. Burnham, Christopher W. Farnsworth, Jennie H. Kwon, for the CDC Prevention Epicenters Program

**Affiliations:** 1 Department of Medicine, Division of Infectious Diseases, Washington University School of Medicine, St. Louis, MO, USA; 2 Department of Pathology & Immunology, Washington University School of Medicine, St. Louis, MO, USA

## Abstract

**Objective:**

To measure SARS-CoV-2 anti-nucleocapsid (anti-N) antibody seropositivity among healthcare personnel (HCP) without a history of COVID-19 and to identify HCP characteristics associated with seropositivity.

**Design:**

Prospective cohort study from September 22, 2020, to March 3, 2022.

**Setting:**

A tertiary care academic medical center.

**Participants:**

727 HCP without prior positive SARS-CoV-2 PCR testing were enrolled; 559 HCP successfully completed follow-up.

**Methods:**

At enrollment and follow-up 1–6 months later, HCP underwent SARS-CoV-2 anti-N testing and were surveyed on demographics, employment information, vaccination status, and COVID-19 symptoms and exposures.

**Results:**

Of 727 HCP enrolled, 27 (3.7%) had a positive SARS-CoV-2 anti-N test at enrollment. Seropositive HCPs were more likely to have a household exposure to COVID-19 in the past 30 days (OR 7.92, 95% CI 2.44–25.73), to have had an illness thought to be COVID-19 (4.31, 1.94–9.57), or to work with COVID-19 patients more than half the time (2.09, 0.94–4.77). Among 559 HCP who followed-up, 52 (9.3%) had a positive SARS-CoV-2 anti-N antibody test result. Seropositivity at follow-up was associated with community/household exposures to COVID-19 within the past 30 days (9.50, 5.02–17.96; 2.90, 1.31–6.44), having an illness thought to be COVID-19 (8.24, 4.44–15.29), and working with COVID-19 patients more than half the time (1.50, 0.80–2.78).

**Conclusions:**

Among HCP without prior positive SARS-CoV-2 testing, SARS-CoV-2 anti-N seropositivity was comparable to that of the general population and was associated with COVID-19 symptomatology and both occupational and non-occupational exposures to COVID-19.

## Introduction

Since the start of the coronavirus disease 2019 (COVID-19) pandemic, healthcare personnel (HCP) have been at risk for exposure to severe acute respiratory syndrome coronavirus 2 (SARS-CoV-2) in occupational and non-occupational settings.^1,2^ Early in the pandemic, this risk was exacerbated by staffing and personal protective equipment (PPE) shortages and lack of widespread availability of SARS-CoV-2 vaccines.^3–6^ Despite increased distribution of SARS-CoV-2 mRNA vaccines through vaccination campaigns, nosocomial outbreaks among patients and HCP persisted, notably during the Omicron variant wave in 2021.^7,8^

As community transmission increased with subsequent variants, HCP COVID-19 rates rose and exacerbated the existing burdens on the healthcare system.^9^ While later SARS-CoV-2 strains exhibited lesser virulence compared to pre-vaccination era strains, COVID-19 continued to be a danger to frontline HCP in the post-vaccination era.^7,8,10^ After introduction of the SARS-CoV-2 mRNA vaccines, vaccination status emerged as a protective factor against both community and HCP infection with COVID-19 within 6 months.^11–15^ However, there is a paucity of evidence specifically on U.S. HCP infection rates during later waves of SARS-CoV-2 variants and on occupational and non-occupational risk factors for COVID-19 among HCP (other than vaccination status) in the post-vaccine era when compared to the pre-vaccine era^16–19^.

Reducing SARS-CoV-2 transmission has been further complicated by asymptomatic infection and pre-symptomatic infections, presenting a challenge for occupational health interventions.^20^ Previous analyses have demonstrated that a significant fraction of COVID-19 transmission is attributable to asymptomatic infection, which evades symptom-triggered testing.^21^ Thus, understanding non-symptom based characteristics associated with COVID-19 among HCP remains important for the control of SARS-CoV-2. The objectives of this study were to measure the rates of SARS-CoV-2 anti-nucleocapsid antibody (anti-N) seropositivity in HCP with no history of COVID-19 in both the pre-vaccine and post-vaccine eras of the pandemic. Anti-N seropositivity was a proxy for prior infection because all major SARS-CoV-2 vaccines stimulate anti-spike protein (anti-S) antibody production, whereas SARS-CoV-2 infection stimulates both anti-S and anti-N production.^22^ Characteristics associated with anti-N seropositivity among these HCP throughout the pandemic were also identified.

## Methods

### Setting and participants

This was a prospective observational cohort study examining SARS-CoV-2 anti-N antibodies in HCP who provided care for COVID-19 patients, worked with COVID-19 patient specimens, or worked in non-clinical spaces on the medical campus, but had no personal history of COVID-19. This study recruited participants from staff of the Washington University in St. Louis School of Medicine (WUSM), an affiliated tertiary care academic medical center (Barnes-Jewish Hospital, BJH), and an affiliated pediatric hospital (St. Louis Children’s Hospital, SLCH). The study protocols were reviewed and approved by the Washington University in St. Louis Human Research Protection Office (IRB# 202008145 and 202104067). All participants provided informed consent prior to specimen and data collection. Participants were enrolled in one of two cohorts: one prior to rollout of SARS-CoV-2 mRNA vaccines, and the other after vaccine rollout.

HCP ≥ 18 years of age who were employed at WUSM, BJH, or SLCH and who worked with patients with COVID-19 or SARS-CoV-2 specimens were eligible to participate. HCP who reported active symptoms of COVID-19 at the time of enrollment or a prior positive SARS-CoV-2 test were excluded. Potential participants were recruited by research staff visits in hospital units and study advertisements posted in staff areas and on the WUSM Volunteer for Health website.

### Survey and specimen collection

Participants completed two study visits: an enrollment visit and a follow-up visit, which was scheduled 30-185 days after the enrollment visit. Enrollment visits were conducted between September 22, 2020 and October 10, 2021. Follow-up visits were conducted between December 1, 2020 and March 3, 2022.

**Figure 1.**
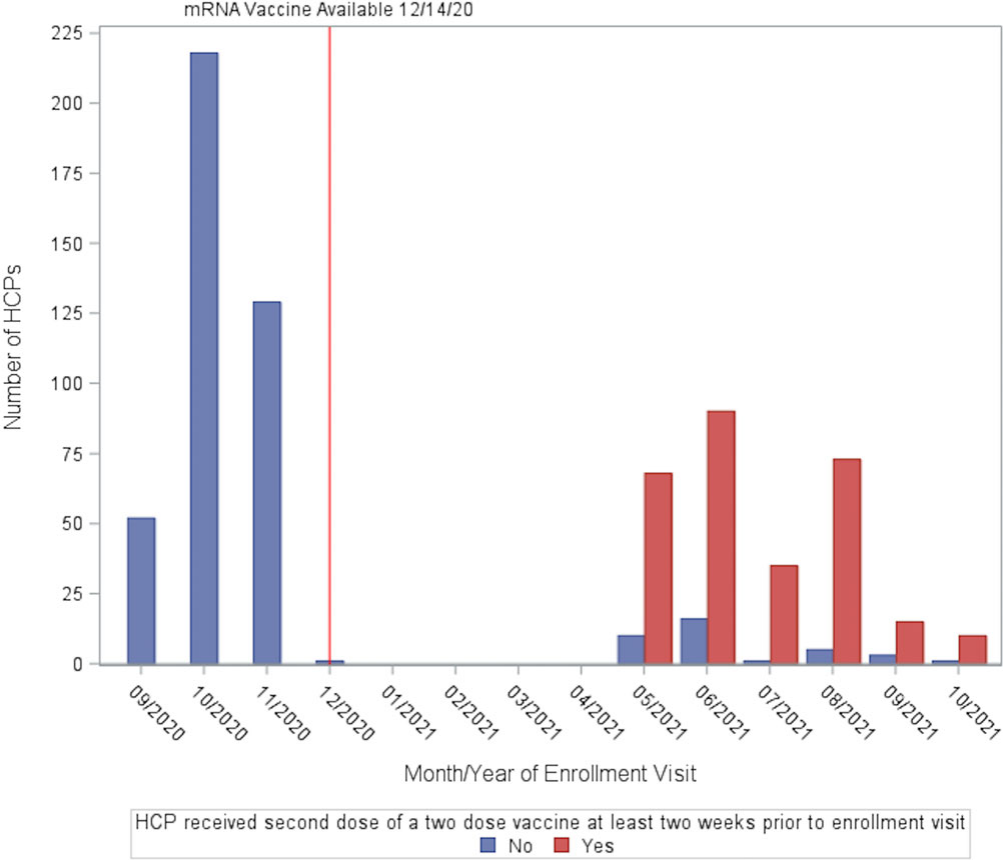
Rates of full vaccination against SARS-CoV-2 among HCP across the study period. Vaccines became widely available for healthcare personnel starting December 14, 2020.

At the time of enrollment, participants completed a survey on demographics, employment, and pre-existing medical conditions. At both study visits, participants provided information about SARS-CoV-2 exposures, social distancing behaviors, travel and social history, and symptoms of COVID-19. mRNA vaccines against SARS-CoV-2 became available to our HCP population on December 14, 2020. Vaccination status at follow-up was documented for all participants, and vaccination status at enrollment was documented for HCP enrolled after vaccines were available. Rates of vaccination before and after December 14, 2020 are shown in Figure 1.

### Antibody testing

At both enrollment and follow-up visits, blood specimens were obtained for SARS-CoV-2 antibody testing. Anti-nucleocapsid (anti-N) protein antibodies were analyzed, as anti-spike protein antibodies (anti-S) are induced by most commercial vaccines and are not specific for prior infection.^22^ Blood specimens were collected into a 10 mL K2 EDTA tube and maintained at room temperature for up to 8 hours before processing. Specimens were centrifuged and plasma aliquots stored for up to 4 days at 4°C before anti-N testing. Anti-N testing was performed on the Abbott SARS-CoV-2 IgG assay (Abbott, Abbott Park USA) according to manufacturer recommendations on an Abbott Architect i2000. The assay is qualitative but reports a signal to cutoff (S/CO) relative to the calibrator. A result of ≥ 1.4 S/CO is considered a positive result.^23^

### Statistical analyses

Participants were grouped by the primary study outcome of seropositivity at enrollment. Chi square and Fisher’s exact tests were used to examine associations between anti-N test results and HCP characteristics as well as SARS-CoV-2 exposure history. Odds ratios (OR) and 95% confidence intervals (CI) were estimated using univariate logistic regression. The same analysis was also performed based on seropositivity at follow-up. For our multivariate analysis, initially variables with *p* < 0.1 in the bivariate analysis were included in the model. Variables were removed in a backwards stepwise manner with *p* < 0.05 as the threshold for retention, and potential multicollinearity of independent variables was assessed using variance inflation factors. Statistical analyses were performed in SAS version 9.4 (Cary, NC), with *p* < 0.05 considered statistically significant.

## Results

### Characteristics associated with SARS-CoV-2 anti-N seropositivity at enrollment

A total of 727 participants completed an enrollment visit, 559 (77%) of whom completed a follow-up visit. The median age of participating HCP was 35 (interquartile range [IQR] 30-47), and the cohort was predominately female and white (Table 1). The majority of participating HCP were involved with direct patient-care and employed on inpatient wards. Most HCP participating in this study reported using disposable surgical masks at work, but 205 (28.2%) reported using N-95s instead of or in addition to surgical masks. The two most common comorbidities among participants were seasonal allergies and BMI ≥ 30 (Table 1).

**Table 1.** Baseline demographics for all HCP enrolled

	Frequency n = 727 (%)
**Demographics**	
Age, Median [IQR]	35 [30, 47]
Female	517 (71.1)
Race	
Asian	79 (10.9)
Black	47 (6.5)
White	597 (82.1)
Other^a^	16 (2.2)
Hispanic ethnicity	21 (2.9)
**Comorbidities**	
Seasonal allergies	350 (48.1)
BMI ≥ 30	103 (14.2)
Cerebrovascular disease	40 (5.5)
Use of immunosuppressive medications	34 (4.7)
Pregnancy	29 (4.0)
Diabetes	22 (3.0)
Lung disease	24 (3.3)
Cancer	18 (2.5)
Smoking	14 (1.9)
Neurologic condition	10 (1.4)
Other comorbidity^b^	72 (9.9)
**Department**	
Medical/Surgical Wards/Specialties	323 (44.4)
Pediatrics	132 (18.2)
Emergency Services	73 (10.0)
Pathology/Pharmacy/Radiology/Laboratory	62 (8.5)
Intensive Care Unit	58 (8.0)
Hospital staff services	13 (1.8)
Other^c^	66 (9.1)
**Job role**	
Direct care provider^d^	500 (68.8)
Other care role^e^	63 (8.7)
Non-care role^f^	160 (22.0)
Missing	4 (0.5)
**Commute method**	
Drive a car alone	635 (87.3)
Walk or bike	72 (9.9)
Other transportation method^g^	54 (7.4)
**Type of mask worn at work**	
Surgical/Isolation (disposable)	622 (85.6)
N-95	205 (28.2)
Cloth	148 (20.4)
Other^h^	6 (0.8)

a Other race includes: Alaska Native, Ashkenazi, Brown, Filipino, Middle Eastern, Native American, Native Hawaiian, Pacific Islander, and South American.

b Other comorbidities include: Adrenal insufficiency, anxiety, attention deficit disorder, attention-deficit/hyperactivity disorder, autoimmune disease, blood disorder, chronic granulomatous disease, chronic kidney disease, chronic urticaria, connective tissue disorder, depression, diabetes insipidus, gastrointestinal conditions, gout, heart disease, history of solid organ transplant, human immunodeficiency virus with CD4 < 200, hyperlipidemia, hypersomnolence, hypothyroidism, liver disease, migraine, mild asthma, narcolepsy, nasal polyps, osteoarthritis, polycystic ovarian syndrome, and sarcoidosis.

c Other departments include: Academic services, billing, biology, child development, and library services.

d Direct care roles include: Advance practice nurse, medical student, nurse, nurse practitioner, physician, and physician assistant.

e Other care roles include: Emergency medical technician, genetic counselor, medical assistant, nursing student, occupational therapist, paramedic, patient care technician, physical therapist, and respiratory therapist.

f Non-care roles include: Administration, child care provider, dietician, dining services, environmental services personnel, facilities personnel, lab personnel, pharmacist, PhD research associate, professor, research support personnel, social worker, speech therapist, and teacher.

g Other transportation methods include: Bus, carpool with coworker, carpool with family member or friend, and train.

h Other mask types include: KF94, KN95, PAPR.

At enrollment, 27 (3.7%) enrolled HCP had a positive SARS-CoV-2 antibody test. There were no significant differences in demographics between SARS-CoV-2 seropositive and seronegative HCP (Table 2); however, seropositive HCP were more likely to have had a household exposure to COVID-19 in the past 30 days (OR 7.92, 95% confidence interval 2.44–25.73) or to work with COVID-19 patients more than half the time (2.09, 0.94–4.77). Social distancing practices and masking practices were not significantly different between seropositive and seronegative HCP. Seropositive HCP were more likely to have had an illness that they thought was COVID-19 (4.31, 1.94–9.57); however, these HCP reported no history of a positive SARS-CoV-2 test at the time of enrollment (Table 2).

**Table 2.** HCP symptomatology and exposures at enrollment in SARS-CoV-2 anti-N positive versus negative individuals

	Reactive antibody test result (n = 27)	Non-reactive antibody test result (n = 700)	OR (95% CI)	*P* value
**Demographics**				
Age greater than 50	7 (25.9)	146 (20.9)	1.33 (0.55, 3.20)	0.53
Female	21 (77.8)	496 (70.9)	1.44 (0.57, 3.62)	0.44
Non-white race	5 (18.5)	135 (19.3)	0.95 (0.35, 2.56)	0.92
**Occupational characteristics**				
Patient care job role^a^	21 (77.8)	542 (77.9)	0.99 (0.39, 2.51)	0.99
Known, specific COVID-19 exposure at work in the past 30 days	6 (22.2)	63 (9.0)	2.87 (1.12, 7.39)	0.028
Contact with known or suspected COVID-19 patients at work more than half the time	10 (37.0)	152 (21.7)	2.09 (0.94, 4.66)	0.07
Practice social distancing when at work and not involved in immediate patient care less than half the time	11 (40.7)	328 (46.9)	0.77 (0.35, 1.69)	0.51
Wear a face mask while at work less than half the time	1 (3.7)	24 (3.4)	1.08 (0.14, 8.27)	0.94
**Non-occupational characteristics**				
Household member known or suspected of having COVID-19 in the past 30 days	4 (14.8)	15 (2.1)	7.92 (2.44, 25.73)	0.004
Other COVID-19 exposure outside of work (excluding sick household member) in the past 30 days	0 (0.0)	14 (2.0)	undefined	1.00
Practice social distancing when in public less than half the time	1 (3.7)	100 (14.3)	0.23 (0.03, 1.71)	0.15
Wear a face mask while in public less than half the time	1 (3.7)	69 (10.0)	2.85 (0.38, 21.21)	0.18
Traveled within past 30 days	5 (18.5)	181 (25.8)	0.64 (0.24, 1.72)	0.38
Attended a large gathering in past 30 days^b^	13 (48.1)	390 (55.7)	0.74 (0.34, 1.59)	0.44
Ate indoors at a restaurant in past 30 days	14 (51.8)	276 (39.4)	1.65 (0.77, 3.57)	0.20
Ate outdoors at a restaurant in past 30 days	16 (59.3)	324 (46.3)	1.69 (0.77, 3.69)	0.19
Visited a medical office in past 30 days	10 (37.0)	319 (45.6)	0.70 (0.32, 1.56)	0.38
Visited a store in past 30 days	26 (96.3)	679 (97.0)	0.80 (0.10, 6.21)	0.83
Visited other public location in past 30 days^c^	17 (63.0)	372 (53.1)	1.50 (0.68, 3.32)	0.32
**Symptoms**				
Had illness thought to be COVID-19 since February 2020	11 (40.7)	96 (13.7)	4.31 (1.94, 9.57)	<0.001

a Patient care roles include: Advance practice nurse, emergency medical technician, genetic counselor, medical assistant, medical student, nurse, nurse practitioner, nursing student, occupational therapist, paramedic, patient care technician, physical therapist, physician, physician assistant, and respiratory therapist.

b Large gatherings include: Amusement park, bar, concert, funeral, gathering of family or friends, graduation, protest, religious services, school, sporting event, trivia night, wedding, and work conference.

c Other public locations include: Bank, Department of Motor Vehicles office, dry cleaner, hair salon, hiking trail, laundromat, library, massage parlor, polling place, post office, vet office, and yoga studio.

### Characteristics associated with SARS-CoV-2 anti-N seropositivity at follow-up

**Figure 2.**
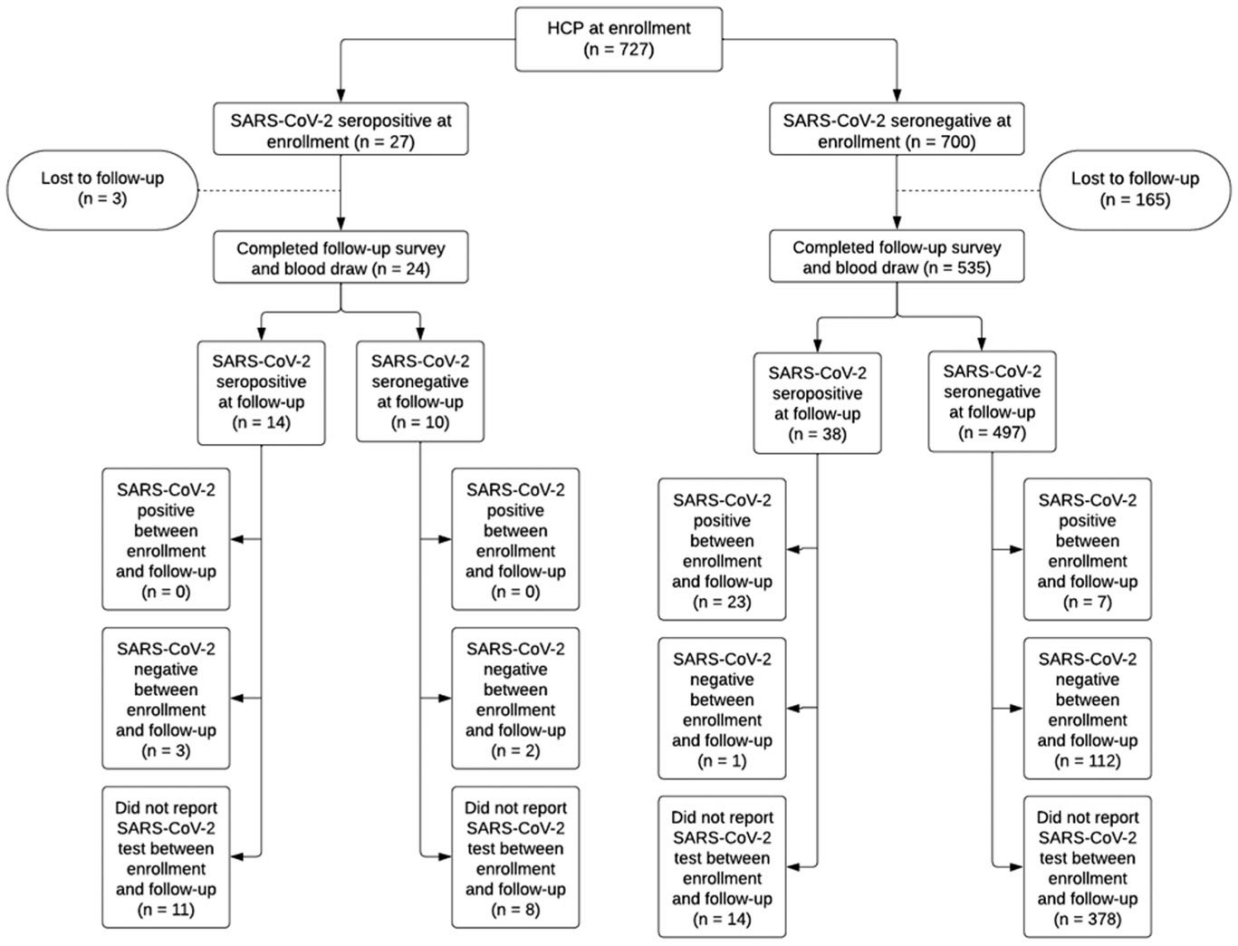
SARS-CoV-2 anti-N seropositivity shifts between enrollment and follow-up.

559 participants were re-evaluated at a follow-up visit 33-185 days from the enrollment visit. At follow-up, 52 (9.3%) of HCP had a positive SARS-CoV-2 anti-N antibody test result. Between enrollment and follow-up, 38 HCP (73% of seropositive HCP at follow-up) converted from seronegative to seropositive, and 10 HCP (37% of seropositive HCP at enrollment) converted from seropositive to seronegative. Additionally, 37 HCP reported having a positive SARS-CoV-2 test (either PCR or antigen) between enrollment and follow-up, 29 (78%) of whom had a reactive antibody test at follow-up. 16 HCP (42%) who seroconverted from negative at enrollment to seropositive at follow-up reported a positive SARS-CoV-2 test (either PCR or antigen) between study visits. 155 HCP reported a negative SARS-CoV-2 PCR test between enrollment and follow-up, 5 (3.2%) of whom had a reactive antibody test at follow-up. These relationships are depicted in the cohort flow chart in Figure 2. There was a lesser association between seropositivity and workplace exposure to COVID-19 patients at follow-up than at enrollment (1.80, 0.92–3.52). Additionally, seropositive HCP were more likely to work with COVID-19 patients more than half the time, although with a less robust association when compared to enrollment (1.50, 0.80–2.78) (Table 3).

**Table 3.** HCP symptomatology and exposures at follow-up 1–6 months from enrollment in SARS-CoV-2 anti-N positive versus negative individuals

	Reactive antibody test result (n = 52)	Non-reactive antibody test result (n = 507)	OR (95% CI)	*P* value
**Occupational characteristics**				
Known, specific COVID-19 exposure at work in the past 30 days	13 (25.0)	81 (16.0)	1.80 (0.92, 3.52)	0.08
Contact with known or suspected COVID-19 patients at work more than half the time	16 (30.8)	118 (23.3)	1.50 (0.80, 2.78)	0.21
Practice social distancing when at work and not involved in immediate patient care less than half the time	21 (40.4)	222 (43.8)	1.15 (0.64, 2.06)	0.63
Wear a face mask while at work less than half the time	2 (3.8)	21 (4.1)	1.08 (0.25, 4.74)	1.00
**Non-occupational characteristics**				
Household member known or suspected of having COVID-19 in the past 30 days	23 (44.2)	39 (7.7)	9.50 (5.02, 17.96)	<0.001
Other COVID-19 exposure outside of work (excluding sick household member) in the past 30 days	9 (17.3)	34 (6.7)	2.90 (1.31, 6.44)	0.009
Traveled within past 30 days	11 (21.1)	144 (28.4)	0.67 (0.34, 1.34)	0.26
Attended a large gathering in past 30 days	20 (38.5)	260 (51.3)	0.59 (0.33, 1.07)	0.08
Ate indoors at a restaurant in past 30 days	23 (44.2)	192 (37.9)	1.31 (0.73, 2.31)	0.36
Ate outdoors at a restaurant in past 30 days	7 (13.5)	141 (27.8)	0.40 (0.18, 0.92)	0.03
Visited a medical office in past 30 days	18 (34.6)	255 (50.3)	0.52 (0.29, 0.95)	0.031
Visited a store in past 30 days	49 (94.2)	489 (96.4)	0.60 (0.17, 2.11)	0.43
Visited other public location in past 30 days^a^	29 (55.8)	289 (57.0)	0.95 (0.54, 1.69)	0.86
**COVID-19 testing and symptoms**				
Had illness thought to be COVID-19 since enrollment survey was completed	25 (48.1)	53 (10.4)	8.24 (4.44, 15.29)	<0.001
**Had test (PCR or antigen) for COVID-19 since enrollment survey was completed**				
No/I’m not sure	18 (34.6)	347 (68.4)	reference	
Yes, test was positive	29 (55.8)	8 (1.6)	69.88 (27.99, 174.47)	<0.001
Yes, test was negative	5 (9.6)	150 (29.6)	0.64 (0.23, 1.76)	<0.001
**COVID-19 vaccine status**^**b**^				0.74
Fully vaccinated	31 (59.6)	328 (64.7)	reference	
Not vaccinated	11 (21.1)	88 (17.4)	1.32 (0.64, 2.74)	0.46
Partially vaccinated	7 (13.5)	75 (14.8)	0.99 (0.42, 2.33)	0.73

a Other public locations include: Bank, car dealership, dry cleaner, hair salon, hotel, laundromat, library, mechanic, medical spa, museum, nail salon, post office, and vet office.

b One employee in the reactive group was missing a response to this question.

SARS-CoV-2 anti-N seropositivity at follow-up was strongly associated with community exposure to COVID-19 and household exposure to COVID-19 (9.50, 5.02–17.96; 2.90, 1.31–6.44) (Table 3). SARS-CoV-2 seropositive HCP were less likely to have attended a large gathering, eaten outdoors at a restaurant, or visited a medical office in the 30 days prior to follow-up. As at enrollment, seropositive HCP were more likely to report having had an illness believed to be COVID-19 (8.24, 4.44–15.29). However, HCP seropositive at follow-up were more likely to have had a positive SARS-CoV-2 PCR and/or antigen test between their enrollment and follow-up study visits than HCP who were seronegative at follow-up (Table 3). Among HCP who reported having had an illness thought to be COVID-19 between enrollment and follow-up, 92.4% sought SARS-CoV-2 testing. Of those who sought testing, 31 (42.4%) tested positive, 22 (71.0%) of whom also had positive anti-N testing at follow-up. Overall, of the 38 newly seropositive HCP at follow-up, 29 (76.3%) had also had a positive interim SARS-CoV-2 test. There was no association between SARS-CoV-2 anti-N seropositivity and vaccination status at follow-up (Table 3).

Using data from the follow-up survey and blood draw, logistic regression was performed to determine risk factors associated with a positive SARS-CoV-2 antibody test result in a multivariate analysis (data not shown). The remaining significant risk factors in the final model were household exposure and illness thought to be COVID-19, in keeping with our findings in the univariate analysis.

## Discussion

This study sought to examine rates of SARS-CoV-2 serologic anti-N antibodies across two years in HCP who cared for patients with COVID-19, handled specimens containing SARS-CoV-2, or worked in the medical campus but had no known history of COVID-19. At enrollment, overall SARS-CoV-2 anti-N seropositivity was 3.7% and was associated with having had symptoms consistent with COVID-19, as well as exposure to COVID-19 in both non-occupational and occupational settings. Overall SARS-CoV-2 anti-N seropositivity at follow-up in our cohort was 9.3%. Household and community COVID-19 exposures were more strongly associated with SARS-CoV-2 anti-N seropositivity than occupational exposures at enrollment and follow-up. SARS-CoV-2 vaccination status was collected at follow-up, as HCP enrolled prior to December 2020 did not have access to vaccines (Figure 1). In this cohort, there was a low SARS-CoV-2 anti-N seropositivity rate: thus, it was underpowered to detect the vaccine’s protective effects against SARS-CoV-2 infection in HCP that have been noted in several clinical trials.^11,24,25^

Overall, our cohort demonstrated lower or comparable SARS-CoV-2 anti-N seropositivity rates among HCP without prior confirmed COVID-19 than in other HCP cohorts.^15,26^ However, it is difficult to compare our cohort’s seropositivity rates to data sets examining “asymptomatic” SARS-CoV-2 anti-N seropositive HCP in other studies, as several HCP in our cohort endorsed previously experiencing COVID-19 symptoms without obtaining testing. In the St. Louis region during the early phases of the pandemic, only 20% of cases were identified by diagnostic RT-PCR testing due to low surveillance testing rates, high rates of symptomatic infection, and issues with testing accessibility.^27^ Thus, some HCP in our cohort may not have been able to obtain SARS-CoV-2 testing, despite having symptoms, and may not have been identified as cases. These individuals would have met our study’s inclusion criteria at enrollment and could thus have contributed to our demonstrated seropositivity rate.

Our study found an increase in SARS-CoV-2 anti-N seropositivity between enrollment and follow-up. This may be related to the increase in community transmission in 2021 with the spread of new SARS-CoV-2 variants, as well as increased duration of exposure at follow-up when compared to enrollment.^28–30^ 11 HCP who converted to SARS-CoV-2 anti-N seropositive between enrollment and follow-up did not report having a positive SARS-CoV-2 test during this period. These cases may not have been tested or confirmed due to the decreased virulence among later variants of SARS-CoV-2.^26^ The likelihood of seropositivity may have also increased at follow-up simply due to general increase in seroprevalence.^31^

Interestingly, 10 HCP (37%) converted from SARS-CoV-2 anti-N seropositive at enrollment to seronegative at follow-up. SARS-CoV-2 anti-N seroreversion in other cohorts has been noted at rates from 5.0% to 38% among HCP (i.e., not just those without a prior positive SARS-CoV-2 PCR test).^32–34^ Previous studies have found lower severity of infection to be a predictor of SARS-CoV-2 anti-N seroreversion, which aligns with our findings of a higher seroreversion rate in HCP who did not have a prior positive SARS-CoV-2 PCR test but were seropositive nonetheless.^32–34^ The discrepancy between previously reported rates and observed rates in our cohort is likely due to our cohort’s smaller sample size, but may reflect geographic differences in SARS-CoV-2 immunogenicity, host antibody production, and sensitivity of serologic testing.

We also examined characteristics associated with SARS-CoV-2 anti-N seropositivity in HCP without history of COVID-19 at both time points. The overall SARS-CoV-2 anti-N seropositivity was low, thus making it underpowered to detect a difference due to behavioral changes (social distancing and mask use, Table 2). Exposure to household members with COVID-19 was associated with SARS-CoV-2 anti-N seropositivity at both enrollment and follow-up, which has been documented.^35–37^ Interestingly, the association between anti-N seropositivity and workplace COVID-19 exposures reached clinical but not statistical significance at follow-up; this may be a consequence of attrition, increased transmission in the community, or decreased transmission in the workplace due to infection prevention practices. Having had an illness thought to be COVID-19 was highly correlated with SARS-CoV-2 anti-N seropositivity, suggesting that self-assessment of infection remained a strong predictor of infection. The majority (92%) of those HCP at follow-up who believed they had had COVID-19 did seek testing between enrollment and follow-up. 76% of HCP with new SARS-CoV-2 anti-N seropositivity at follow-up also had a positive SARS-CoV-2 test and believed to have had COVID-19 in the interim. Symptom screening early in the COVID-19 pandemic was considered unreliable given the high burden of asymptomatic carriers and non-specificity of symptoms.^38,39^ Our data suggest that these tools may still be effective in the HCP population and are consistent across time periods with multiple variants and implementation of vaccines.

Our study had several limitations. As noted previously, although we enrolled 727 HCP, our cohort was underpowered to conduct a formal risk factor analysis associated with COVID-19 due to lower seropositivity rate at enrollment. Changes at follow-up relative to baseline could thus be related to attaining adequate power at follow-up (due to higher overall seroprevalence rates), although this is also confounded by attrition and increased vaccination rates from enrollment to follow-up. Different HCP had different periods of follow-up after their enrollment visit (33–185 days after enrollment). This would affect each HCP’s risk of SARS-CoV-2 anti-N seropositivity, persistence of SARS-CoV-2 anti-N seropositivity, and vaccination status. Additionally, the study period occurred over the course of two years during the COVID-19 pandemic. During this time, changes in public policy, virus strains, and workplace safety regulations occurred that would give each HCP a different risk factor profile.^5,6^ This is particularly significant due to HCP vaccination status which differed based on availability of SARS-CoV-2 mRNA vaccines (and, consequently, the effects of boosters and hybrid immunity) at different points in the pandemic, and because of the magnitude of the effect that the vaccines had in reducing SARS-CoV-2 transmission.^14,40^ Extended follow-up periods also increased the likelihood that HCP would undergo COVID-19 testing, the results of which may have biased their self-assessment of prior symptomatology at follow-up. Furthermore, our data set was predominantly composed of young, white, female HCP employed at a large, Midwestern academic center, which may not be representative of all healthcare settings.

## Research transparency and reproducibility

Contributing data and code may be shared at the discretion of the corresponding author.
